# Anti-neutrophil cytoplasmic antibody associated vasculitis in patients with rheumatoid arthritis

**DOI:** 10.1186/s12882-022-02788-6

**Published:** 2022-04-22

**Authors:** Haiting Wu, Yiyun Lu, Rongrong Hu, Wei Ye, Yubing Wen, Jianfang Cai, Hang Li, Xuemei Li

**Affiliations:** 1 Division of Nephrology, Peking Union Medical College Hospital, Chinese Academy of Medical Sciences & Peking Union Medical College, Beijing, 100730 China; 2 Department of Internal Medicine, Peking Union Medical College Hospital, Chinese Academy of Medical Sciences & Peking Union Medical College, Beijing, 100730 China; 3 Division of Nephrology, Fuwai Hospital, Chinese Academy of Medical Sciences & Peking Union Medical College, Beijing, 100037 China

**Keywords:** Anti-neutrophil cytoplasmic antibody associated vasculitis, Rheumatoid arthritis, Glomerulonephritis

## Abstract

**Background:**

Anti-neutrophil cytoplasmic antibody (ANCA)-associated vasculitis (AAV) may coexist with rheumatoid arthritis (RA). However, it is unclear whether the manifestations of AAV with and without coexisting RA are similar. This observational study aimed to investigate the clinicopathological manifestations of AAV with coexisting RA and to explore potential predictors for identifying AAV superimposed on RA.

**Methods:**

Patients with both AAV and RA were identified by searching our hospital database and the literature. Data including age, sex, clinical manifestation, laboratory tests, renal pathology, and therapeutic regimens were retrieved. To assess the difference in clinical features and renal pathology between AAV patients with and without RA, we conducted 1:4 matched case-control studies.

**Results:**

A total of 47 patients were identified, 15 from our hospital and 32 from the literature, and 33 (70.2%) were women. AAV was diagnosed later than RA in 83.0% of the patients and manifested as microscopic polyangiitis (MPA) in 78.7% of the patients. The kidney was the most frequently involved extra-articular organ (74.5%), followed by the lung (51.1%), and skin (8.5%). Patients with both AAV and RA were more likely to be asymptomatic (26.7% vs 3.3%, *p =* 0.013) than those with isolated AAV. However, they did not differ in other clinicopathological features. In RA patients, those with ANCA associated glomerulonephritis, were more likely to have decreased renal function at renal biopsy as opposed to those with primary glomerulonephritis.

**Conclusions:**

AAV can coexist with RA. In this coexistence, AAV usually develops after RA, is more likely to be asymptomatic, and manifests predominately as MPA with renal involvement. Thus, we should remain vigilant to superimposed AAV on RA.

**Supplementary Information:**

The online version contains supplementary material available at 10.1186/s12882-022-02788-6.

## Key points

- RA patients may have a greater tendency to develop AAV
- AAV superimposed on RA may be asymptomatic with elevated serum creatinine.

## Background

Anti-neutrophil cytoplasmic antibody (ANCA) -associated vasculitis (AAV) is a group of small vessel vasculitis including microscopic polyangiitis (MPA), granulomatosis with polyangiitis (GPA), and eosinophilic granulomatosis with polyangiitis. It can cause severe damage to organs, especially kidneys. Early diagnosis of AAV is essential for the preservation of organ function [1]. It has been reported that AAV may coexist with rheumatoid arthritis (RA) [2]. ANCA-associated glomerulonephritis patients with RA appear to have smoldering clinical features and may result in late referral from rheumatologists to nephrologists and therefore have a poor prognosis [3]. However, research on patients with the coexistence of AAV and RA is limited, and most studies are case reports. It is unclear whether there are any differences between AAV with and without coexisting RA. We therefore reviewed cases with these coexisting diseases in our hospital and in the literature, and summarized the clinical manifestations in these patients. We also attempted to identify the clinicopathological characteristics of AAV patients superimposed on RA by comparing them to those with AAV without RA. In consideration of the poor prognosis of AAV-associated glomerulonephritis, we analyzed the risk factors for AAV renal involvement in RA patients by renal biopsy in our center to timely identify these patients.

## Methods

### Study subjects

Patients with both AAV and RA in the Peking Union Medical College Hospital from January 2000 to December 2018 were included in this study. AAV was diagnosed based on the criteria proposed by the Chapel Hill Conference [4]. The diagnosis of RA complied with the 2010 ACR/EULAR RA classification criteria [5]. Cases before 2010 were reassessed and re-diagnosed. We also searched for cases of both AAV and RA published in English or Chinese between January 2000 and December 2018 in MEDLINE, EMBASE, and Chinese databases including China National Knowledge Infrastructure, Wanfang Database, and VIP Database. The keyword for RA was “rheumatoid arthritis”. The keywords for AAV were “anti-neutrophil cytoplasmic antibody associated vasculitis”, “anti-neutrophil cytoplasmic antibody associated nephritis”, “anti-neutrophil cytoplasmic antibody”, “small vessel vasculitis”, “rapidly progressive glomerulonephritis”, “microscopic polyangiitis”, “granulomatosis with polyangiitis”, “Wegener’s granulomatosis”, “Churg-Strauss syndrome”, “eosinophilic granulomatosis with polyangiitis” and “allergic granulomatous angiitis” (Supplementary Fig. 1). We excluded patients exposed to potential AAV-inducing drugs such as tumor necrosis factor-α inhibitors, penicillamine, and propylthiouracil [6].

### Study design

We pooled all the patients from our center and the literature to demonstrate the clinical manifestations of AAV coexisting with RA. In order to assess clinical features and renal pathology of AAV coexisting with RA, we 1:4 matched patients with both AAV and RA in our center with those having isolated AAV by sex, age, AAV subtypes, and presence of renal involvement and renal pathology. We then evaluated the association between RA and ANCA-associated glomerulonephritis in renal-biopsied patients in our hospital. In order to identify potential predictors for ANCA-associated glomerulonephritis in RA patients, we compared the clinical features between renal-biopsied RA patients with ANCA-associated glomerulonephritis and primary glomerulonephritis.

Data on sex, age, serology, clinical manifestation, interval between diagnosis of RA and AAV, renal pathology, and therapeutic regimens, if applicable, were collected retrospectively from medical records or the literature. Tubulointerstitial chronic index, assessed by the percentage of tubular atrophy and interstitial fibrosis in the total area of the cortical tubulointerstitium was classified into four grades:1: ≤25%, 2: 26–50%, 3: 51–75%, and 4: >75%. Histopathology of ANCA associated glomerulonephritis was re-classified according to the criteria proposed in 2010 [7].

### Statistical analysis

Continuous variables are presented as mean ± standard deviation or median (interquartile range) and compared using the Student t-test or Mann-Whitney U test as appropriate. Categorical variables are presented as n (%) and compared using the chi-square test or Fisher’s exact test. Logistic regression was employed to assess the association between RA and ANCA associated glomerulonephritis in renal-biopsied patients by adjusting for sex and age. All statistical tests were 2-sided, with significance defined as *p* < 0.05. All statistical analyses were performed using the SPSS 23.0 (IBM).

## Results

### Clinical features of AAV coexisting with RA

#### Patients with both AAV and RA in our center

In total, 15 patients with both AAV and RA were identified in our center (Table 1). Of these patients, all were categorized as MPA, 9 (60.0%) were women, and 9 (60.0%) had renal pathology and were diagnosed with ANCA associated glomerulonephritis. MPA was diagnosed 5.0 (2.0–20.0) years later than RA. At diagnosis of MPA, the patients were on average 54 ± 17 years old and the kidney (86.7%) was most frequently involved, followed by the lung (53.3%). Kidney involvement manifested as elevated serum creatinine (80.0%), proteinuria (86.7%), and hematuria (86.7%). All patients received glucocorticoids and 14 (93.3%) cases received immunosuppressants, cyclophosphamide in 13 and mycophenolate mofetil in one, to treat AAV. During a median follow-up of 12 (1–60) months, 4 of 14 patients progressed to end-stage renal disease (ESRD) and 2 died due to rupture of an abdominal aortic aneurysm and pulmonary infection, respectively.

**Table 1:** Clinical characteristics of patients with AAV and coexisting RA

Characteristics	Our center (***n*** = 15)	Literature (***n*** = 32)	Total (***n*** = 47)
**Female**	9 (60.0%)	24 (75.0%)	33 (70.2%)
**Age at diagnosis of AAV (ys)**	54 ± 17	50 ± 15	51 ± 15
**ANCA serology**^**a**^			
P-ANCA or myeloperoxidase -ANCA	14 (93.3%)	24 (75%)	38 (80.9%)
C-ANCA or proteinase 3-ANCA	2 (13.3%)	6 (18.8%)	8 (17.0%)
negative	0 (0.0%)	2 (6.3%)	2 (4.3%)
**Vasculitis diagnosis**			
Microscopic Polyangiitis	15 (100.0%)	22 (68.8%)	37 (78.7%)
Granulomatosis with polyangiitis	0 (0.0%)	10 (31.3%)	10 (21.3%)
**Chronological order of diseases**			
Rheumatoid arthritis first	13 (86.7%)	26 (81.3%)	39 (83.0%)
ANCA associated vasculitis first	0 (0.0%)	4 (12.5%)	4 (8.5%)
Contemporaneous	2 (13.3%)	2 (6.3%)	4 (8.5%)
**Interval between diagnosis of RA and AAV (ys)**	5.0 (2.0–20.0)	6.5 (1.6–12.0)	5.0 (2.0–12.0)
**Organs involved**			
Kidney	13 (86.7%)	22 (68.8%)	35(74.5%)
Lung	8 (53.3%)	16 (50.0%)	24(51.1%)
Skin	1 (6.7%)	3 (9.4%)	4(8.5%)
Nose	0 (0.0%)	2 (6.3%)	2(4.3%)
Nervous system	1 (6.7%)	0 (0.0%)	1 (2.1%)
**Renal manifestations**			
	***n*** **= 15**	***n*** **= 19**	***n*** **= 34**
Serum creatinine (umol/L)	164 (106–471)	292 (148–352)	282(132–379)
	***n*** **= 15**	***n*** **= 17**	***n*** **= 32**
24-h urine protein(g/d)	1.56(0.38–4.79)	0.94 (0.50–3.10)	1.25(0.50–3.40)
	***n*** **= 15**	***n*** **= 32**	***n*** **= 47**
Rapidly progressive glomerulonephritis	2(13.3%)	4(12.5%)	6(12.8%)
**Therapy for RA**	***n*** **= 12**	***n*** **= 29**	***n*** **= 41**
Gold compounds	1 (8.3%)	5 (17.2%)	6(15.6%)
Methotrexate	1 (8.3%)	10(34.5%)	11(26.8%)
Leflunomide	4 (33.3%)	2(6.9%)	6(14.6%)
**Therapy for AAV**	***n*** **= 15**	***n*** **= 31**	***n*** **= 46**
Glucocorticoid	1 (6.7%)	4 (12.9%)	5(10.9%)
Glucocorticoid plus cyclophosphamide	13 (86.7%)	16 (51.6%)	29(63.0%)
Glucocorticoid plus other immunosuppressants	1 (6.7%)	9 (29.0%)	10(21.7%)
None	0 (0.0%)	2 (6.5%)	2(4.3%)
**Prognosis**	***n*** **= 14**	***N*** **= 31**	***n*** **= 45**
Improved	8 (57.1%)	22 (71.0%)	30(66.7%)
End Stage Renal Failure	4 (28.6%)	7 (22.6%)	11(24.4%)
Death	2 (14.3%)	2 (6.5%)	4(8.9%)

*Abbreviations:ANCA* anti-neutrophil cytoplasmic antibody, *AAV* ANCA-associated vasculitis, *RA* rheumatoid arthritis

^a^One patient in our center was both myeloperoxidase -ANCA and proteinase 3-ANCA positive

Cases from the literature had missing data related to some parameters. For a parameter with missing data, we gave a specific number of participants with data on this parameter

#### Patients with both AAV and RA from our center and the literature

We further identified 32 cases with both AAV and RA from the literature (Table 1). Patients with suspected drug-induced AAV were excluded. We pooled these cases with those from our center [3, 8–24]. The pooled 47 patients were aged 51 ± 15 years at diagnosis of AAV and 33 (70.2%) were women. AAV was diagnosed before, at the same time, and after the diagnosis of RA in 4 (8.5%), 4 (8.5%), and 39 (83.0%) cases, respectively. Of these patients, 37 (78.7%) were categorized as MPA and 10 (21.3%) as GPA. The kidney was the most frequently involved extra-articular organ, followed by the lung and skin. Renal involvement manifested as hematuria, moderate proteinuria and renal insufficiency. At diagnosis of AAV, the patients with renal involvement had a median creatinine level of 282 (132–379) μmol/L. Glucocorticoids with or without immunosuppressants were prescribed to treat AAV in 95.7% of patients. Glucocorticoid plus cyclophosphamide was the first-choice regimen (63.0%), followed by glucocorticoid plus other immunosuppressants (21.7%), and glucocorticoid alone (10.9%). In our center, cyclophosphamide was given intravenously at a dose of 0.4–0.6 g per week during hospitalization and then orally after discharge. However, in the literature, cyclophosphamide was given orally, intravenously, or both. During a median follow-up of 12 months, 30 (66.7%) of 45 patients showed improved renal function, 11 (24.4%) developed ESRD, and the remaining 4 patients (8.9%) died.

### Differences in clinical features and renal pathology between AAV patients with and without RA in our center

As all of the patients with AAV and coexisting RA in our center had MPA, we restricted the comparison of clinicopathological features between MPA with and without RA. Between January 2000 and December 2018, 411 MPA patients without RA were identified in our center. Patients with both MPA and RA were marginally younger than those with isolated MPA (54 ± 17 vs 61 ± 14 years, *p* = 0.052), but they did not differ in sex distribution (60% female vs 52.2% male, *p* = 0.55).

We then matched 15 patients with both MPA and RA to 60 patients with isolated MPA by age, sex, and presence of renal involvement and renal biopsy (Table 2). Patients with both MPA and RA were more likely to be asymptomatic (*p* = 0.013) and less likely to have fever (*p* = 0.010) at diagnosis of MPA as opposed to those with isolated MPA. However, the two groups did not differ in other clinical features and renal pathology, including weight loss, lung involvement, presence of rapidly progressive glomerulonephritis, severity of proteinuria, baseline estimated glomerular filtration rate (eGFR), Birmingham Vasculitis Activity Score (BVAS), and improvement in renal function after 6 months.

**Table 2:** Clinical characteristics of MPA patients with or without RA

	With RA(*n* = 15)	Without RA^a^(*n* = 60)	*P*
**Clinical symptoms**			
None	4(26.7%)	2(3.3%)	.013
Fever	4(26.7%)	39(65.0%)	.010
Weight loss	5(33.3%)	27(45.0%)	.56
**Organ involvement**			
Lung	8(53.3%)	34(56.7%)	1.00
rapidly progressive glomerulonephritis	3(20.0%)	25(41.7%)	.15
Other organs	9(60.0%)	40(66.7%)	.76
**Laboratory tests**			
Hemoglobin(g/L)	92 ± 26	97 ± 22	.43
ESR (mm/h)	72 ± 45	70 ± 41	.86
C-creative protein (mg/L)	3.08 (1.05,7.92)	3.35 (1.25, 13.94)	.60
Albumin(g/L)	32 ± 7	31 ± 6	.51
24-h urine protein (g)	1.56(0.38–4.79)	1.10(0.53–2.45)	.81
eGFR [ml.min^−1^. (1.73 m^2^)^−1^]	34.8 ± 28.4	41.5 ± 30.6	.45
**BVAS**	14 ± 5	17 ± 8	.27
**Normalization of SCr or decrease of SCr by 50% at 6th month**	4(28.6%,n = 14)	22(40.0%,*n* = 55)	.43

*Abbreviations:ESR* erythrocyte sedimentation rate, *eGFR* estimated glomerular filtration rate, *BVAS* Birmingham Vasculitis Activity Score, *SCr* serum creatinine

^a^Patients with both AAV and RA in our center were 1:4 matched with patients with isolated AAV adjusted by sex, age, AAV type, and presence of renal involvement and renal biopsy

Laboratory tests were recorded at diagnosis of AAV

In order to assess the difference in renal pathology between patients with both MPA and RA and those with isolated MPA, we further matched the 9 patients with both pathology-proved ANCA associated glomerulonephritis and RA to 36 patients with isolated pathology-proved ANCA associated glomerulonephritis by age and sex (Table 3). The two groups had renal histopathology consistent with pauci-immune crescentic glomerulonephritis and did not differ in the percentage of global glomerulosclerosis, cellular crescents, and histopathological classes recommended by Berden et al. [7].

**Table 3:** Renal histopathology in MPA patients with or without RA

	With RA(*n* = 9)	Without RA^a^(*n* = 36)	*P*
Percentage of normal glomeruli	24.3 ± 10.8%	27.9 ± 22.7%	.64
Percentage of cellular crescent glomeruli	23.1 ± 18.7%	34.0 ± 18.4%	.10
Percentage of global sclerotic glomeruli	37.0 ± 23.5%	26.9 ± 24.0%	.26
Tubulointerstitial chronic index	1.9 ± 0.9	1.6 ± 0.9	.46
Histopathological classification^b^			
Focal class	1 (11.1%)	7 (19.4%)	.51
Crescent class	1 (11.1%)	9 (25.0%)	
Mixed class	3 (33.3%)	13 (36.1%)	
Sclerotic class	4(44.4%)	7 (19.4%)	

^a^Renal-biopsied patients with both MPA and RA in our center were 1:4 matched with patients with isolated MPA adjusted by sex and age

^b^ANCA-associated glomerulonephritis was classified according to the histopathological classification proposed in 2010 [7]

### ANCA-associated glomerulonephritis in RA patients undergoing renal biopsy in our center

Between January 2000 and December 2018, 55 RA patients underwent renal biopsy in our center, 9 had ANCA associated glomerulonephritis, 23 had renal impairment caused by other systemic conditions such as lupus nephritis, diabetic glomerulopathy, hepatitis B virus associated glomerulonephritis, and drug-induced renal injury, and the remaining 23 patients had primary glomerulonephritis (glomerulonephritis without apparent secondary causes other than RA). Of these 23 patients, membranous nephropathy and IgA nephropathy were the top two pathological types (11 and 5 patients, respectively). RA patients with ANCA associated glomerulonephritis, as opposed to those with primary glomerulonephritis, tended to have lower eGFR and hemoglobin and higher levels of serum albumin at biopsy (Table 4). Multivariable logistic regression analysis revealed that an eGFR< 30 ml.min^− 1^ (1.73 m^2^)^− 1^ at renal biopsy was associated with ANCA associated glomerulonephritis in renal-biopsied RA patients (OR 25.13 [1.07–592.06], *p* = 0.046) adjusted for age, sex, serum albumin and hemoglobin.

**Table 4:** Clinical characteristics of RA patients with pathology-proved ANCA-associated glomerulonephritis and primary glomerulonephritis

	ANCA-associated glomerulonephritis (*n* = 9)	Primary glomerulonephritis (*n* = 23)^a^	*P*
Age (ys)	49 ± 21	51 ± 11	.75
Female	7 (77.8%)	18 (78.3%)	1.00
Duration of rheumatoid arthritis (ys)	5 (4–20)	9(1–14)	.49
Hemoglobin (g/L)	97 ± 19	120 ± 21	.009
ESR (mm/h)	66 ± 42	71 ± 31	.70
eGFR [ml.min^−1^.(1.73 m^2^)^−1^]	24.4 ± 15.1	84.8 ± 21.2	<.001
Serum albumin (g/L)	35 ± 7	27 ± 9	.018
24-h urine protein (g)	1.73 (0.37,4.47)	4.20 (2.31,9.07)	.07
Hematuria	9 (100.0%)	19 (82.6%)	.30
Treated with immunosuppressants	5 (55.6%)	12 (52.2%)	1.00

*Abbreviations:ANCA* anti-neutrophil cytoplasmic antibody, *ESR* erythrocyte sedimentation rate, *eGFR* estimated glomerular filtration rate

^a^RA patients with renal biopsy results were included. Among them, renal injuries secondary to other systemic diseases such as lupus nephritis and diabetic nephropathy were excluded

Laboratory tests were recorded at renal biopsy

## Discussion

RA is largely an inflammatory disease limited to joints, whereas AAV is a systemic autoimmune disease usually with multi-organ destructive processes and devastating outcomes. It has been reported that AAV can coexist with RA [3, 8–24]. This coexistence may not be entirely coincidental, as evidenced by facts such as ANCA positivity in 20% of RA patients [25], a positive association between ANCA titer and rapid joint destruction in early RA [26], the presence of myeloperoxidase-ANCA in synovial fluid of RA patients [27], and AAV sharing susceptibility loci with RA [28, 29]. Our finding, that renal-biopsied patients with RA were more likely to have AAV than those without RA, adds new evidence to the inherent association between RA and AAV.

In our center, as well as in the literature, AAV usually occurred after RA in patients with both RA and AAV [10–24]. This may partially be ascribed to the successful inhibition of RA occurrence by intense immunosuppression in the treatment of AAV. Alternatively, disease modifying antirheumatic drugs used for RA may also change the typical manifestations of AAV to a less extra-renal and extra-articular presentation, with a lower frequency of rapidly progressive glomerulonephritis, and more chronicity in renal pathology, as demonstrated in our and Kurita’s study [3]. This deviation may hamper or delay our recognition of AAV in RA patients and finally result in a poor outcome.

Renal involvement is a major feature of AAV and is not rare in RA patients. In RA patients with biopsy-proved primary glomerulonephritis, membranous nephropathy and IgA nephropathy were the top two histopathological patterns as revealed by our and previous studies [30]. RA patients with ANCA-associated glomerulonephritis were more likely to have renal dysfunction than those with primary glomerulonephritis. Therefore, ANCA should be assessed in RA patients with renal dysfunction.

Our study revealed an overwhelming predominance of MPA in AAV patients when AAV coexists with RA. This is consistent with findings in the literature. However, the underlying mechanism of this phenomenon is unclear.

There are some limitations in this study. First, because this was a retrospective study and the patients enrolled were highly selected, there was inevitable bias, which may have undermined the robustness of the study findings. Second, most of the patients from our center underwent renal biopsy. As such, the findings of the present study may be biased by the variable indications of renal biopsy for different underlying renal disease. Third, given the small sample in the study, care should be taken regarding generalization of the results of the study.

In conclusion, AAV may not be as rare as we thought in patients with RA. In patients with both AAV and RA, AAV usually developed after RA, was more likely to be asymptomatic, and manifested as MPA with renal involvement. We recommend routine assessment to determine the presence of AAV in RA patients with renal involvement, especially in those with renal dysfunction.

## Supplementary Information

**Additional file 1.**

## Data Availability

The data used to support the findings of this study are available from the corresponding author upon request.

## Abbreviations

AAV: ANCA associated vasculitis
ANCA: Anti-neutrophil cytoplasmic antibody
BVAS: Birmingham Vasculitis Activity Score
eGFR: Estimated glomerular filtration rate
ESR: Erythrocyte sedimentation rate
ESRD: End stage renal disease
GPA: Granulomatosis with polyangiitis
MPA: Microscopic polyangiitis
RA: Rheumatoid arthritis
SCr: Serum creatinine
