# Supplementary material for: Anti-neutrophil cytoplasmic antibody associated vasculitis in patients with rheumatoid arthritis
Source: BMC Nephrol. 2022 Apr 22;23:155. doi: 10.1186/s12882-022-02788-6 (PMC9026933; doi:10.1186/s12882-022-02788-6)
Supplement: Supplementary file 1 — Additional file 1. [file 12882_2022_2788_MOESM1_ESM.docx]

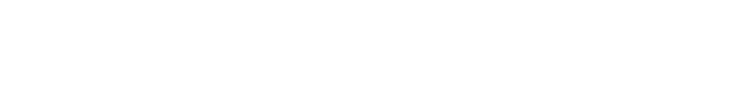


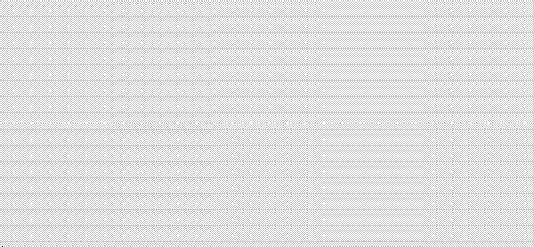

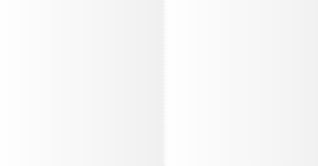

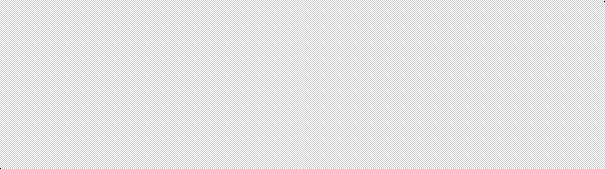

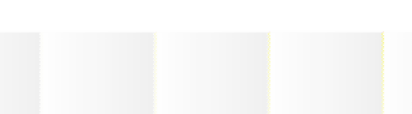

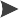

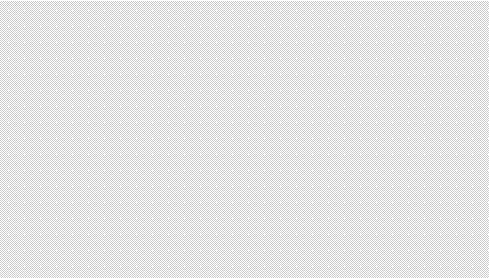

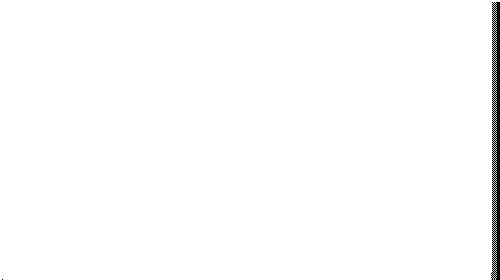

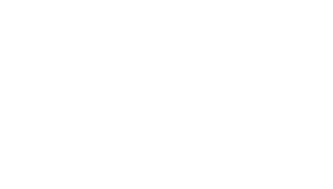

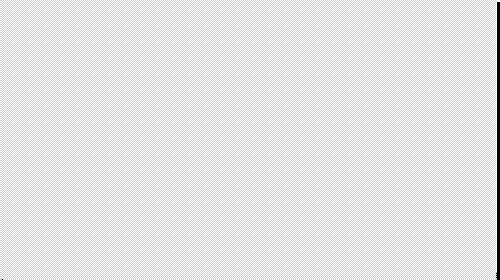

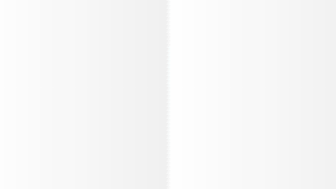

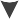

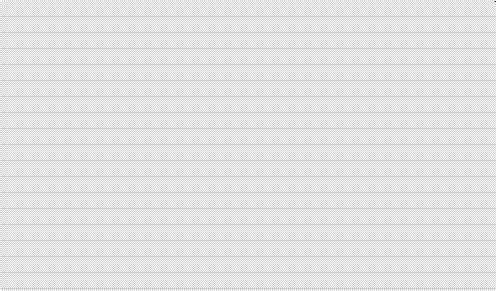

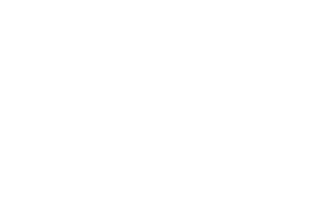

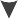

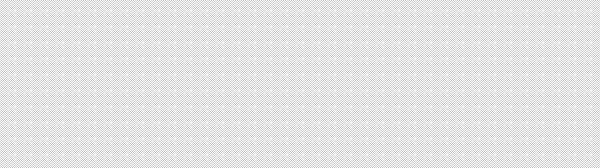

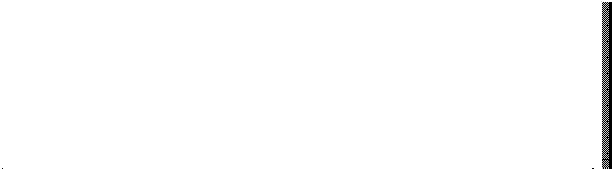

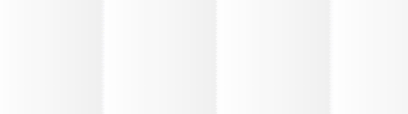

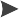

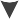

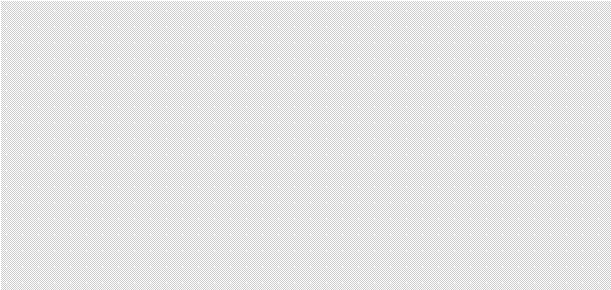

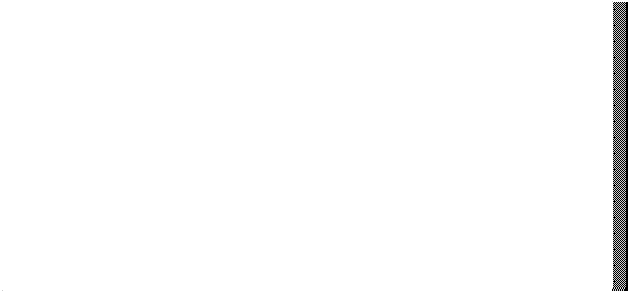

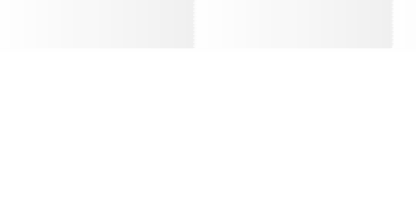

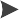

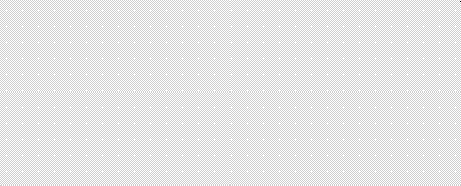

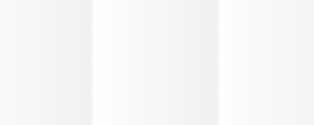

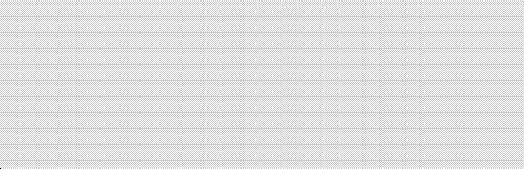

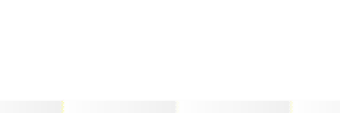

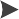

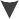


Patients in our hospital:15

Articles included: Medline 14

Embase 1

Wanfang 2

VIP 1

CNKI 0

Patients included:32

Literature to be examined for TNFαinhibitor:

Medline 25

Embase 1

Wanfang 2

VIP 1

CNKI 0

Potential literature for the research Medline 30

Embase 14

Wanfang 2

VIP 2

CNKI 1

Preliminary search results Medline 323

Embase 410

Wanfang 4

VIP 5

CNKI 1

Patients included:47

Patients with TNF αinhibitor history were excluded:17

Excluded for other reasons: Duplication 14

Without full text 2

With other connective tissue diseases or malignancy 3

Dignosis not confirmed 1

Titles or abstracts excluded as had no cases with concurrent ANCA associated vasculitis and rheumatoid arthritis :694
